# Fault Diagnosis for Reducers Based on a Digital Twin

**DOI:** 10.3390/s24082575

**Published:** 2024-04-17

**Authors:** Weimin Liu, Bin Han, Aiyun Zheng, Zhi Zheng

**Affiliations:** College of Mechanical Engineering, North China University of Science and Technology, Tangshan 063210, China; lzhjia@ncst.edu.cn (W.L.); 15613540530@163.com (B.H.); zhengzhi@ncst.edu.cn (Z.Z.)

**Keywords:** digital twin, fault diagnosis, dynamics, reducer, human–computer interaction

## Abstract

A new method based on a digital twin is proposed for fault diagnosis, in order to compensate for the shortcomings of the existing methods for fault diagnosis modeling, including the single fault type, low similarity, and poor visual effect of state monitoring. First, a fault diagnosis test platform is established to analyze faults under constant and variable speed conditions. Then, the obtained data are integrated into the Unity3D platform to realize online diagnosis and updated with real-time working status data. Finally, an industrial test of the digital twin model is conducted, allowing for its comparison with other advanced methods in order to verify its accuracy and application feasibility. It was found that the accuracy of the proposed method for the entire reducer was 99.5%, higher than that of other methods based on individual components (e.g., 93.5% for bearings, 96.3% for gear shafts, and 92.6% for shells).

## 1. Introduction

Reducers are commonly used in various transmission systems, such as robots [1,2,3], cars, rolling mills, and so on. The condition monitoring and fault diagnosis of gearboxes has been attracting considerable attention [4,5,6]. The existing fault diagnosis methods mainly rely on machine learning algorithms [7] and deep learning models [8,9,10,11], where Convolutional Neural Networks (CNNs) [12,13], Long Short-term Memory (LSTM) Networks [14], and Autoencoders [15] have shown good performance in terms of feature extraction, although with application restrictions in some fields.

Facing challenges associated with the translation invariance principle, such as idle neurons with slight image direction or position changes, CNN-based methods require an overwhelming amount of data due to their reliance on backpropagation [16]. Additionally, the pooling layers used in CNNs often result in the loss of valuable information and further disregard the correlations. To overcome these defects, many scholars have made various attempts in their works; for example, Lei et al. [17] conducted a comprehensive review of fault diagnosis methodologies, providing insights into the structure of planetary gearboxes and fixed shaft planetary gearboxes. The distinctive behaviors and fault characteristics of planetary gear systems were also identified and analyzed. In another study, Moslem Azamfar et al. [18] proposed a novel fault diagnosis approach grounded in motor current characteristic analysis. A unique 2D CNN architecture was utilized to seamlessly integrate data from multiple current sensors, enabling direct classification without the need for manual feature extraction. Additionally, Ding et al. [19,20,21] introduced a framework for motor fault diagnosis, addressing issues related to representation learning scalability and the neglect of diverse working conditions. In the same year, a novel continuous learning framework was introduced to address the low efficiency of manual fault detection. Additionally, a method was devised to tackle the limitations of knowledge distillation, leveraging a fusion model based on CNN-Gated Recurrent Units (CNN-GRUs) and incorporating a channel attention mechanism.

LSTM-based networks and their derivatives sequentially process data over time [22]. Considering their natural characteristics, they are susceptible to vanishing gradients, particularly when dealing with long sequences. For extremely long sequences, gradient vanishing reduces the accuracy of the method, together with increasing the computational demand and training time, as has been previously verified.

Regarding Autoencoders, they also demand extensive unlabeled data for effective training [23]. Furthermore, the overfitting caused by noise or outliers in the training data might comprise the generalization capability of the model, leading to potential false alarms or missed detections [24]. Furthermore, the unintuitive high-dimensional hidden representations complicate their interpretation in the troubleshooting process, as well as their outputs. Apparently, their applicability is still limited by these inherent constraints.

Although more methods have been developed for the purpose of fault diagnosis, many studies have arrived at a similar conclusion, focusing on the lack of condition monitoring of parts, single working conditions, and/or analysis based on individual parts instead of the whole system, leading to the possibility of a large deviation between virtual and real signals. For example, a rolling bearing fault diagnosis method based on a digital twin (DT) [25] has been proposed, which presents disadvantages for real-time online analysis and diagnosis. A method leveraging CycleGAN [26] was introduced, and a gear DT model for fault diagnosis was constructed; however, a successful individual gear fault diagnosis might not facilitate further analysis of the whole gearbox. This phenomenon becomes more significant when analyzing complex systems. Undoubtedly, those works have made remarkable contributions in the field, but the proposed approaches often overlook the complex relationships and diversity inherent to the system, resulting in subpar performance in terms of capturing fault characteristics and achieving accurate classification [27]. In addition, single modeling methods relying on a restricted data set may not encompass sufficient integrated fault scenarios.

As a supplement to the mentioned research on such integrated systems, the DT concept was introduced in 2002 and has been developed since then. Compared with the traditional methods, DT supports further systematic analyses due to its high compatibility, interactivity, and convenience of application.

Among the milestones in DT, Grieves [28] first proposed the concept of DTs, which involves creating a mirrored model of a physical entity. DT theory has grown rapidly in recent years. Barricelli et al. [29] provided a summary of the definition of DTs and analyzed their differences. Wang et al. [30] developed a DT model for autoclave systems and improved the prediction capabilities of autoclave failures using numerical data over actual failure data. Aivaliotis et al. [31] integrated a physical model with a DT. Errandonea et al. [32] conducted an in-depth study on the life cycle maintenance phase of DTs. Melesse et al. [33] conducted a systematic literature review to evaluate the utility of DTs in industrial operations. Wright et al. [34] highlighted the differences between models and DT, outlined the advantages of DTs, and suggested future research directions. Lechler et al. [35] explained the relationship between the function and application of DTs. Rasheed et al. [36] summarized the methods, techniques, and model construction of DTs. Bordleau et al. [37] summarized various model-driven engineering techniques in the context of model construction for model solution. Rios et al. [38] conducted a comprehensive review on the modeling of measurement uncertainty in data transfer standards and its relationship to test data in DT models. Karve et al. [39] proposed a construction method for DTs, in order to detect and predict uncertain crack growth for damage detection. In the next year, Andronas et al. [40] discussed the limitations of DT modeling with flexible materials. Matulis et al. [41] reported the development of a 3D printing robotic arm and created a DT model in Unity3D. He et al. [42] applied a DT in intelligent detection robots using multi-sensor data fusion technology. Using a DT model, the integration of virtual and physical entities can be realized, where the collected data facilitate simulation, health monitoring, diagnosis, and maintenance [43,44,45,46].

With the development of DT technology, replication technology presents significant potential and promotes the expansion of technical concepts. DT technology is now widely used in online monitoring and intelligent device diagnosis. Tao et al. [47,48] proposed a 5D-DT model based on the original 3D structure with an added service system and communication connection for fault prediction and health management; however, this model is still a long way from full implementation due to hardware and software limitations. Zong et al. [49] developed a set of multi-robot monitoring systems based on DT technology, which monitor the robot arm’s working state, but not the equipment’s operating state and does not allow for deeper data analysis. Liu et al. [50] highlighted the process of offline data collection for a ship structure bearing monitoring system but did not realize real-time monitoring and diagnosis capabilities. Li et al. [51] presented a condition monitoring method for a gear test bench-based DT using real-time data; however, this method only realizes condition monitoring based on real-time data, and it cannot complete further data mining analysis.

As a supplement to the related research, an innovative method based on DTs was proposed in this work for the considered system, with the expectation that a comprehensive approach could be adopted to construct the reducer, including the effects of temperature and noise. DT and Unity 3D technologies are applied for the online diagnosis and human–computer interaction. The main goals achieved by this work are as follows:

(1) DT technology was applied to model the overall reducer.
(2) A systematic fault diagnosis method was proposed to realize visual displays and online diagnoses of the DT.
(3) The whole analysis under variable speed condition was realized and validated through a practical application.

To supplement DT fault diagnosis, the processes used in this work are as follows:

(1) To achieve high accuracy, a whole model of a reducer is built, and a test platform is established to acquire simulated and real signals under various operating conditions.
(2) In order to enable visualization and online diagnosis, field vibration data are subjected to noise filtering and reduction techniques, thus eliminating interference in the middle and low frequency ranges. The processed data are then integrated into the Unity3D reducer DT fault diagnosis system.
(3) To further validate the method’s feasibility, collaboration with local enterprises is carried out to test its practical implementation in production.

The remainder of this article is organized as follows. Section 2 introduces the construction of the DT system. Section 3 provides an experimental example of the DT fault diagnosis system. Section 4 explains the technical route of human–computer interactions based on DTs. The feasibility of the proposed method is verified and compared with other state-of-the-art methods. Section 5 provides the conclusions of the research.

## 2. Framework of Proposed Method

At present, the primary fault diagnosis categories include bearing issues (e.g., pitting, inner and outer ring cracks, and faults in the rolling body) and gear-related problems (e.g., missing teeth, broken teeth, cracks, and pitting). The absence of teeth dominates in most fields, which lowers transmission efficiency. Moreover, it induces additional vibration and noise, gear movement dysfunction, and corruption of the device’s stability and reliability. Furthermore, it increases gear wear under an uneven load distribution and may lead to premature reducer failure. To address these fault types, after the initial comparison, research on tooth absence faults under four working conditions was conducted with the hope of supplementing the related contributions.

**Figure 1:**
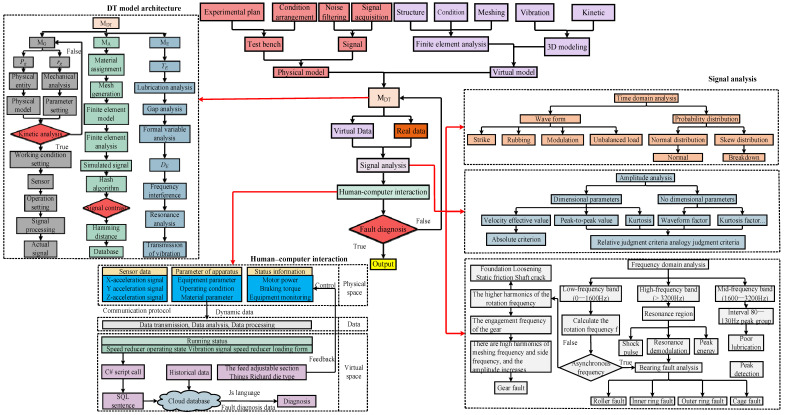
Flowchart depicting the overall study framework.

To begin, the finite element analysis transient dynamics module was applied to construct simulation signals under different working conditions. Then, both real and simulated signals were collected and fed into the human–computer interaction window. In addition, the applicability of the model under multiple working conditions was verified experimentally. Finally, the accuracy of the model was judged according to the hash distance, and further tests were carried out in the context of actual production applications. The overall framework and fault diagnosis process of this work are shown in Figure 1.

### 2.1. Physical Space of DT Model

The construction of physical models plays a crucial role in quality control, physical property analysis, and prediction services. Physical models can be classified into static and dynamic models, where a static physical model quantitatively describes the properties, states, and behaviors, which are solely determined by the entity itself. On the other hand, dynamic physical modeling extends a finite number of nodes in the time domain in order to obtain the state distribution of dynamically changing physical systems.

**Figure 2:**
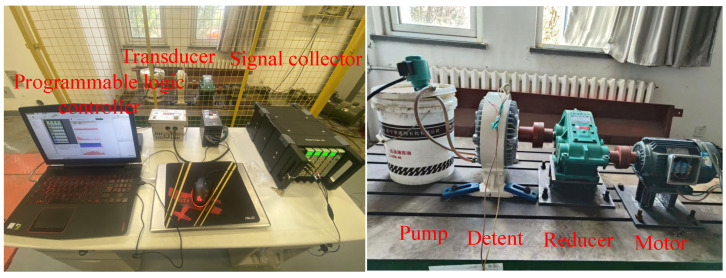
The employed test bench for the reducer.

The physical space parameters include the real-time operating status, parameter performance, sensor information, data sample frequency, and measurement point configuration. Geometric information encompasses shape, size, tolerance, coaxiality, and surface accuracy. Material data are determined according to the reducer material. The motion form is determined based on the field working conditions and loading form. To collect gear failure data, a gear failure test bench was established, which consists of a motor (YE2-90L-4), reducer (JZQ200), magnetic powder brake (FZY400J), PLC, controller (HD800), transducer (FS1000-2R2G/4P), and pump (DB-12A-40W), as depicted in Figure 2. The data were collected using a B&K device with a sampling frequency of 2.56 kHz and a sampling time of 0.2 s. A sensor was placed on the bearing end cover of the reducer to collect vibration data during its operation.

### 2.2. Virtual Space of DT Model

**Figure 3:**
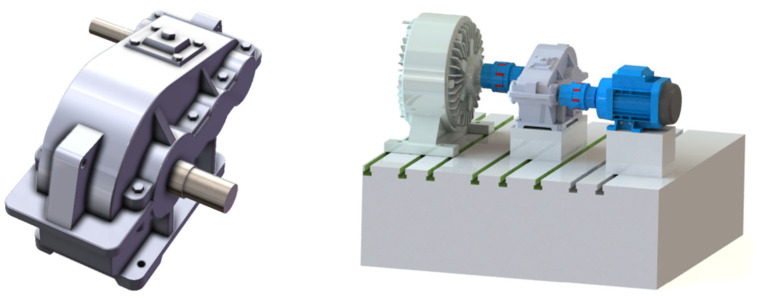
Virtual model of reducer.

**Figure 4:**
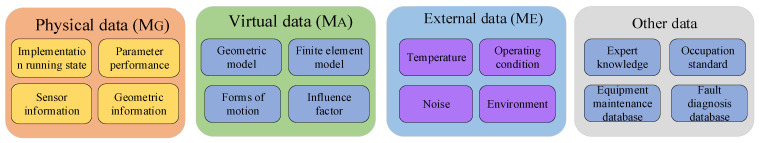
DT model construction.

The virtual space consists of geometric data, along with external effects, where the data are obtained from the output of the analysis model. The virtual model and test bench are shown in Figure 3. The external data include environment, temperature, operating conditions, and noise data. Expert knowledge, industry standards, inferences, equipment maintenance rule bases, and fault diagnosis data can be included as other acknowledged data; see Figure 4.

Virtual modeling encompasses several aspects. First, following the creation of the model, it is saved as a STEP file. AutoCAD 2023 and SolidWorks 2023 software are utilized to create virtual objects and simply simulate their physical properties and behavior. Second, the model is simplified by removing overlapping or irrelevant surfaces, as well as deleting unnecessary lines and structures. ANSYS Discovery 2023 R1 can be utilized to optimize the grid layout and enhance simulation accuracy. Third, the model is imported into HyperMesh 2021 for further grid division. Finally, the data from the virtual sensors are combined to form a convinced data pool. The interactive virtual data and database connection were established using the Unity3D platform. The collected data were analyzed in order to retrieve the data and their definitions.

The dimensions of the considered gear are listed in Table 1.

**Figure 5:**
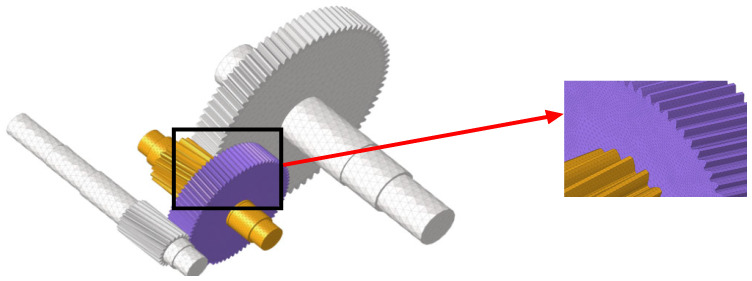
Meshing configuration.

Following these steps, a virtual model of the reducer can be constructed. This method is based on actual data, ensuring precise simulation results. Precise meshing is of high importance, as it allows for accurately representation of geometric shapes and topologies through covering more details and local features. This, in turn, facilitates more accurate physical simulations and dynamic analyses. The completed meshing configuration is shown in Figure 5.

After grid meshing, the material properties were assigned. Accurate material parameters, such as elastic modulus, Poisson ratio, and density, are crucial when describing material behaviors in finite element analysis. In this study, structural steel was used for the reducer, and the parameters are listed in Table 2.

**Figure 6:**
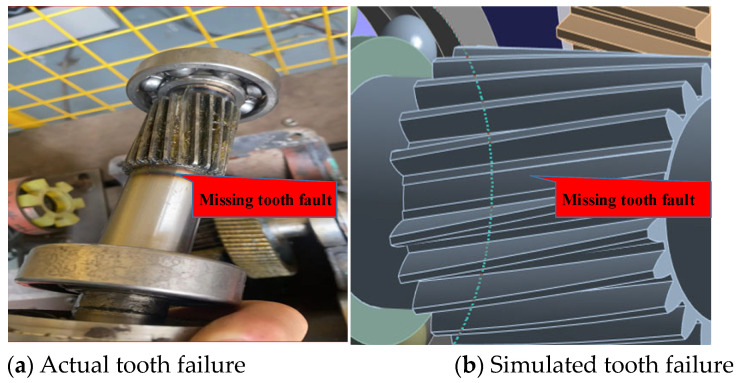
Gear failure in both physical and 3D models.

The model was constructed based on the given parameters, and a missing tooth fault was intentionally created; Figure 6a,b show real and modeled fault samples. To perform a diagnostic analysis, four different working conditions were designed, which were categorized into two loading modes: constant and variable speed.

### 2.3. Twin Space of DT Model

The twin space refers to a combination of physical and virtual spaces that encompass the working conditions and other information of the reducer, in which, the relevant data of the twin system can be optimized and updated. Furthermore, the system is designed to simulate, test, and optimize various real-world conditions and faults. Through digital modeling and simulation, it becomes possible to evaluate the performance of products and obtain accurate predictions. This approach reduces resource consumption through the effective utilization of virtual elements. The DT model depicted in Figure 1 was applied to investigate the vibration patterns under various working conditions, and the modeling process is detailed in the following.

The traditional DT model is shown in Equation (1).

*MO* = {*I*_1_, *I*_2_, *I*_3_, *P*_1_} (1)

Here, *MO* and *P*_1_ represent the traditional DT model of the reducer and effect of the environment, and *I*_1_, *I*_2_, and *I*_3_ represent historical, behavioral data, and device relationships, respectively. 

The update model consists of online, update data, and running features, as established in Equation (2).

*MO_curr_* = {*I*_4_, *I*_5_, *MO*, *P*_1_*^curr^*} (2)

Here, *MO_curr_* is a dynamic update model driven by monitoring data, and *I*_4_, *I*_5_, and *P*_1_*^curr^* represent online data, updated data, and characteristics of the current device, respectively.

The DT model of the reducer was constructed as shown in Equation (3).

*M_DT_* = {*M_G_*, *M_A_*, *M_E_*} (3)

Here, *M_G_*, *M_A_*, and *M_E_* represent the geometric, analytical, and environmental models, respectively.

*M_G_* is employed for the object, encompassing both the dynamic *P_g_* and mechanical model *R_g_*, and can be estimated using Equation (4).

*M_G_* = {*P_g_*, *R_g_*} (4)

The DT model focuses on the vibration and excitation of gear meshing. The dynamic model, *P_g_*, is defined in Equation (5).

*P_g_* = *T_tor_* − *G* × *F_mesh_* + *J* × *θ* (5)

Here, *J*, *θ*, *T_tor_*, *G*, and *F_mesh_* represent the gear moment of inertia, rotation angle, input torque, transfer ratio, and meshing force, respectively. The gear meshing mechanics model *R_g_* in Equation (4) is defined in Equation (6).

*R_g_* = *K* × (*x*_1_ − *x*_2_) + *D* × (*v*_1_ − *v*_2_) + *F_mesh_* (6)

Here, *K*, *x*, *D*, and *v* denote the gear stiffness, displacement, damping coefficient, and velocity, respectively.

The analytical model in Equation (7) is applied to solve the problem, outcome prediction, and inference through the use of various techniques such as Hash algorithms and Hamming distance for signal similarity, while using expert knowledge bases and historical data for supplementary judgment.

*M_A_* = {*A_l_*, *D_is_*, *k_d_*, *h_d_*} (7)

Here, *A_l_*, *D_is_*, *k_d_*, and *h_d_* represent the algorithms, discriminator, knowledge bases, and historical data, respectively.

The vibration of equipment could also be affected by environmental factors such as temperature and noise; therefore, the environmental model in Equation (1) can be addressed using Equation (8):

*M_E_* = {*T_E_*, *D_E_*} (8)

Here, *T_E_* and *D_E_* represent the effects of temperature and noise.

The viscosity, friction, and load of lubricating oil can be influenced by temperature variations, due to the deformation or damage caused by thermal expansion and contraction of internal materials. Consequently, the temperature effect is shown in Equation (9).

*T_E_* = *C* × *exp*(*B*/*T*) + *λ* × *L_0_* × Δ*T* (9)

Here, *C* and *B* represent two empirical parameters; *η*, *T*, *L*, and *λ* represent the dynamic viscosity, temperature, length, and linear thermal expansion coefficient, respectively.

Frequency interference, vibration transfer, and resonance are considered within the effects of noise. Resonance, in particular, potentially results in the amplification of amplitude or destructive vibrations, and described by Equation (10):

(10) $$D_{E}=F_{ex}/2D(\omega_{n}^{2}-\omega_{d}^{2})+\int_{0}^{t}G(t-\tau )a_{n}(\tau )d\tau +F/K\sqrt{\left(1-\omega^{2}/\omega_{0}^{2}{)}^{2}+2(D\times \omega /\omega_{0}^{2}\right)}$$

where *ω_n_*_,_, *ω_d_*, and *F_ex_* represent the noise frequency, natural frequency, and external force, respectively; *a_n_* (*t*) and *G* (*t*) denote the vibration acceleration under noise and the transfer function; and *ω* denotes the intrinsic angular velocity.

## 3. Experimental Verification of the Proposed Model

### 3.1. Experimental Scheme

**Figure 7:**
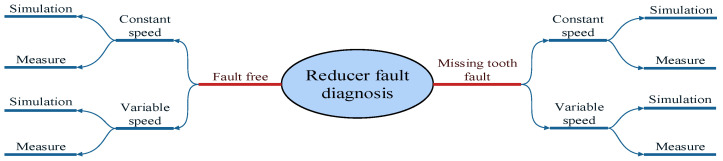
The flow chart of the experiment.

In this experiment, four working conditions were designed to test the performance of the reducer. First, a faultless constant speed test was conducted, where the reducer ran smoothly at 120 rpm. The second was a faultless variable speed test, where the input speed gradually increased over time. The third involved a constant speed test with a missing tooth on the high-speed shaft at 120 rpm. Finally, the simulation of the missing gear fault was performed under a variable speed (which linearly increased with time). Although the reducer works under various working conditions, the four conditions considered in this experiment represent the possible issues encountered in daily operations. Obviously, the faulty and normal states could be distinguished in the experiment. The experimental scheme is shown in Figure 7.

### 3.2. Experimental Boundary Condition

**Figure 8:**
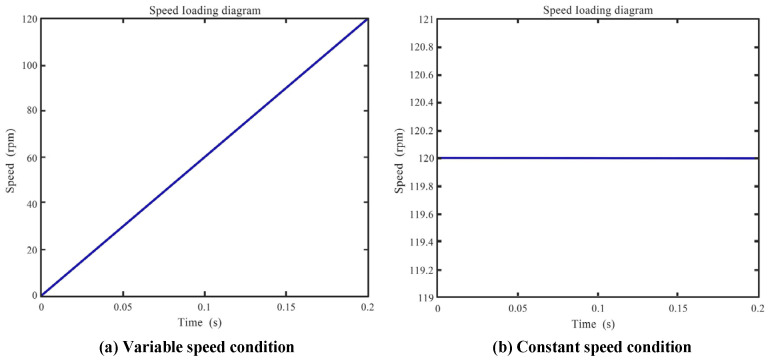
Loading form of variable speed and constant speed.

The program for speed control was written using WPLSoft2.51. To select the PLC mode, the memory address of the special registers (D1062, D1115) could be changed and the mode was set to analog voltage output. After changing the memory address of the special registers D1062 and D1115, the PLC mode was activated for voltage output. Then, the time conversion and the maximum analog output were set in the specific order, with their relationship illustrated in Figure 8. COM1 was selected as the connection port, which was utilized as a mutual port between the laptop and PLC, whose VO0-AG and AI1-GND ports enable internal communication. Inside the PLC, a control loop was formed through connecting the self-locking X_0_ and self-resetting X_1_ ports. Additionally, the inverter was set to analog input control mode.

To control the speed of the reducer, the frequency of the inverter was adjusted, where 30 Hz corresponds to 120 rpm. Next, the load was applied to the brake under the corresponding conditions. Subsequently, a sensor was fixed onto the reducer to acquire data with a sampling frequency of 2.56 kHz. Finally, the faultless and faulty drive shafts were assembled consecutively, in order to collect various forms of data. A total of 32 experiments were conducted, yielding 87 sets of valid data.

### 3.3. Result Analysis

The gear fault diagnosis can be partitioned into three parts. First, the fault is determined through time domain analysis. Second, the severity of the fault is identified based on the amplitude. Finally, the spectrum is used to locate the fault. The specific process is illustrated in Figure 1.

#### 3.3.1. Data Preprocessing

**Figure 9:**
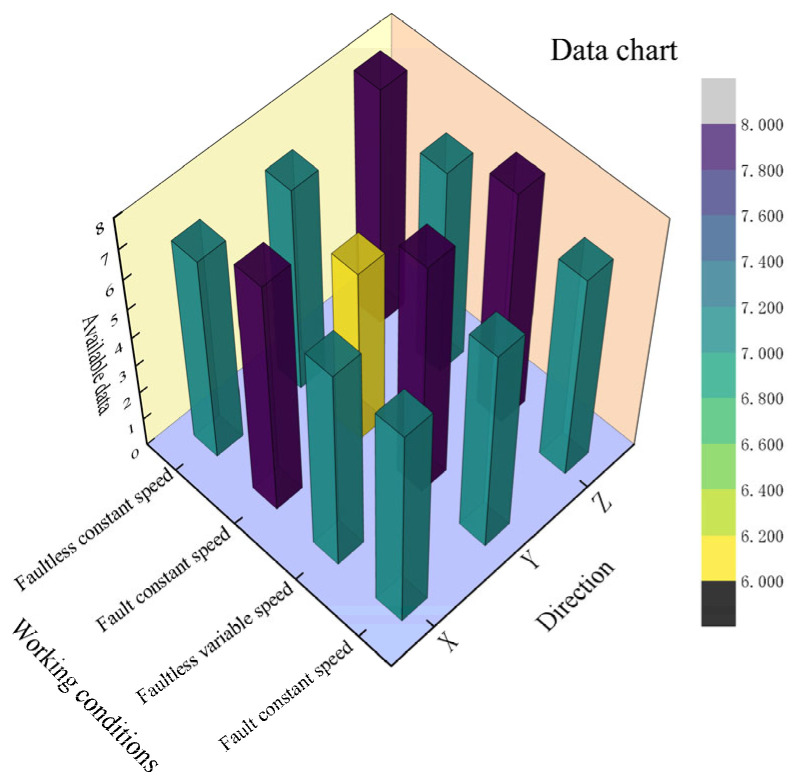
Data statistics.

Preliminary data were collected from the individual eight-repetition experiments for each of the four conditions, which are plotted as 3D bars in this section. Out of 96 sets, a total of 87 sets of valid data were obtained, including X (7 and 8), Y (7 and 6), Z (8 and 7) for faultless and fault constant speed, and X (7 and 7), Y (8 and 7), and Z (8 and 7) for faultless and fault variable speeds. The distribution of the data is shown in Figure 9.

#### 3.3.2. Analysis of Time Domain Signal

**Figure 10:**
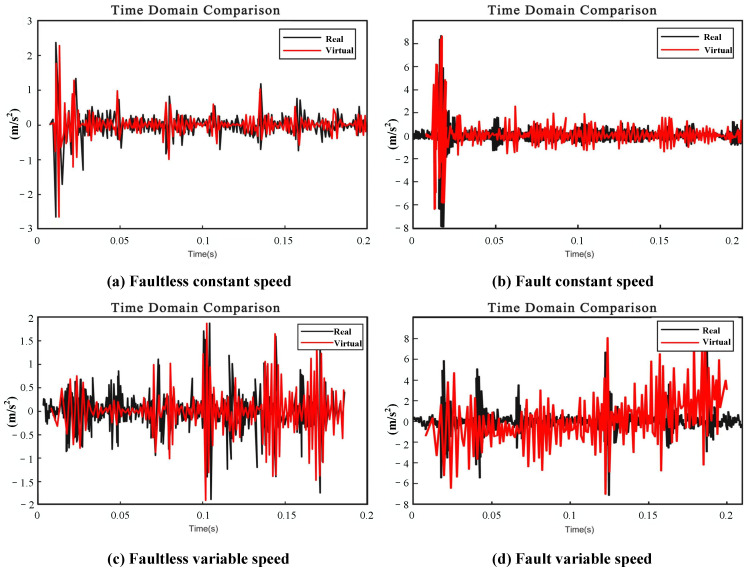
Time domain comparison.

Figure 10 depicts the comparison of the numerical and experimental vibration time domain signals. Figure 10a illustrates the simulated and real vibration time domain comparison of the faultless reducer at a constant working speed of 120 rpm, which demonstrates a periodic change in vibration over time and the vibration state during meshing. The subsequent Figure 10b presents a comparison between the simulated and real signals of the reducer under fault constant speed conditions. Comparing Figure 10a,b, the difference was significant where the missing tooth fault apparently increased the amplitude. Figure 10c compares the simulated and real signals under faultless variable speed conditions. It can be seen that, as the speed increased, the time interval of vibration in the mesh decreased. Comparing Figure 10a,c indicates that the amplitude increases with the speed, aligning with reality. Similarly, the comparison between Figure 10c,d can also be used to monitor the abnormal state according to the change in amplitude.

#### 3.3.3. Analysis of Frequency Spectrum Signal

**Figure 11:**
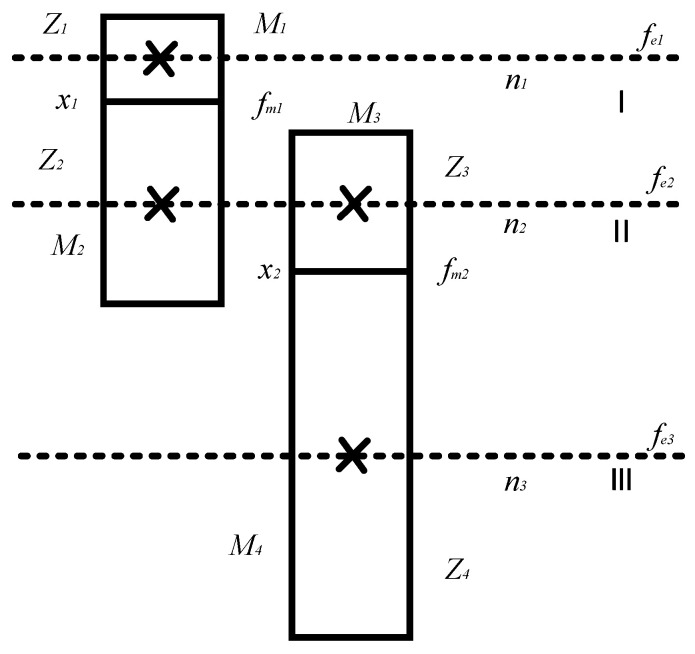
Gear transmission schematic. Here I, II, and III represent the high-speed, intermediate, and output shaft, respectively.

The mechanical model of the gear is depicted in Figure 11. Fault diagnosis was conducted by analyzing the meshing frequency, amplitude of the harmonic component spectrum, amplitude of the side frequency component, main frequency, and distribution difference. In its detailed implementation, a time domain diagram is useful for fault prediction, while spectral domain analysis is popular for the determination of the fault type and location.

The meshing frequency of the gear shaft, the rotation frequency of each gear shaft, and the gear vibration were estimated using Equations (7)–(9), respectively. The obtained values are provided in Table 3.

*f_e_* = (*n*/60) × *Z* (11)

*f_m_* = *f_e_* × *Z* (12)

*M* × *x″* + *D* × *x′* + *K*(*t*) × [*x − e*(*t*)] = (*M*_2_
*− i* × *M*_1_)/*r*_2_ (13)

Here, *n* is the speed of the gear shaft; *f_e_* and *f_m_* represent the rotation frequency and meshing frequency, respectively; and *m* is the mass of each gear shaft.

**Figure 12:**
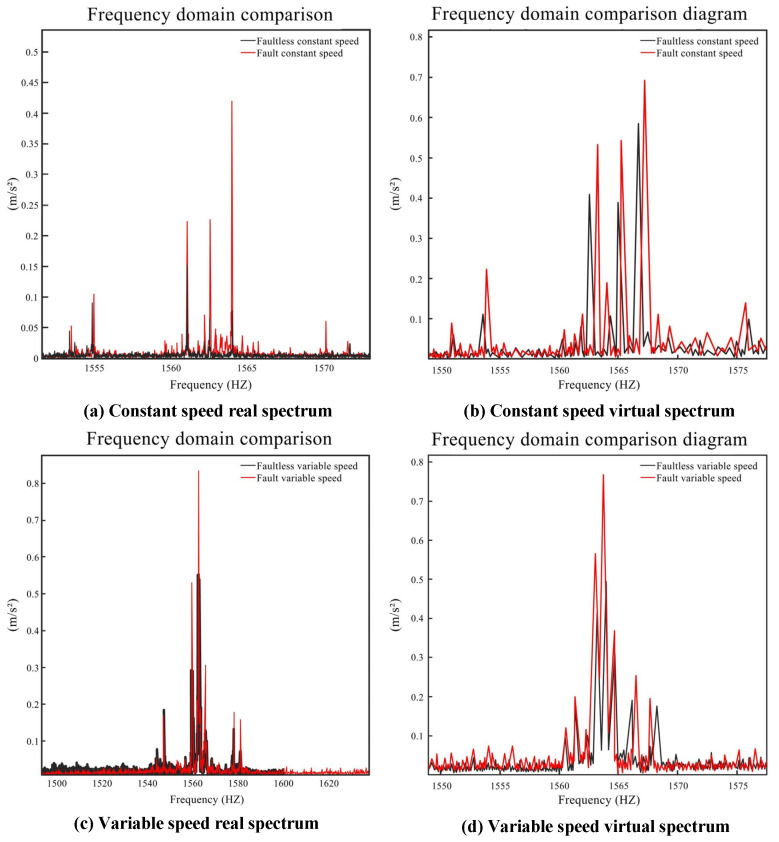
Spectral correlation.

The frequency spectrum diagram of the reducer in Section 3.3.3 was also analyzed, as shown in Table 3, to validate the correctness of the DT model. First, the faultless and fault constant speed cases are compared in Figure 12a. In the spectrum diagram at 1550–1570 Hz, (*f_m_* − *f_e_*, *f_m_* + *f_e_*) can be observed on both sides of the 1562 Hz point. The spectrum reveals that the frequency conversion band had a low amplitude and a relatively flat distribution, suggesting that the reducer had a concentrated defect. The introduction of the missing tooth fault intensified the amplitude of the reducer at the same frequency. The comparison between faultless and fault variable speed conditions is illustrated in Figure 12c, where the amplitude under the fault condition was significantly higher. Finally, the simulation signals of the DT model are displayed in Figure 12b,d. The analysis focused on the mid-frequency band, where the meshing frequency and frequency doubling are clearly visible.

### 3.4. Similarity Test of Time and Frequency Results

To assess the similarity between real and simulated data, the Differential Hash and Hamming distance methods were utilized. First, the Hash algorithm was employed to obtain the Hash values H_1_ and H_2_. Next, the Hamming distance D was obtained according to H_1_ and H_2_. Finally, the similarity was evaluated through a comparison between D and a predetermined threshold. If D is less than or equal to 5, then S_1_ and S_2_ were considered to be similar.

Through employing this approach, the comparison of real and simulated data was brief and efficient. The Hash algorithm provides a concise representation of the data, while the Hamming distance calculation quantifies the dissimilarity between the Hash values. Through comparison with a threshold, the data similarity could be assessed. The Hamming distance threshold is defined by Equation (14).

*d* = (1/2) × (*n_c_* − *k_bit_* + 1) (14)

Here, *d*, *n_c_*, and *k_bit_* represent the minimum Hamming distance, code word length, and number of information bits, respectively. This formula assumes that the code word is constructed from binary symbols, and that each symbol is independent with equal probability. The results are listed in Table 4.

The results showed that the Hamming distance was 1 and similarity was 99.9% and 99.7% under faultless constant and variable speeds. The fault constant speed Hamming distance was 2, with a similarity of 99.6%, and the fault variable speed Hamming distance was 4, with a similarity of 99.3%. The results of four tests were averaged, and the final accuracy was 99.5%. The similarity test results are provided in Table 4.

### 3.5. Result Conclusion and Comparison with Other Fault Diagnosis Methods

The existing fault diagnosis methods for DT reducers primarily rely on the monitoring of an individual component, such as bearings (93.5%), gear shafts (96.3%), or shells (92.6%). To complement the related research, a fault diagnosis approach based on a holistic model was proposed in this study, which obtained an average accuracy rate of 99.5%, providing an alternative for the characterization of the reducer failure state.

#### 3.5.1. Comparison of Image Generation Methods

**Figure 13:**
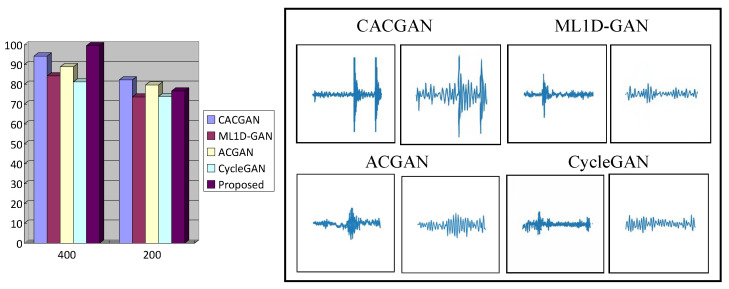
Comparison of image generation methods.

Many scholars have made outstanding contributions in the field of image generation [52]. Therefore, to further illustrate the efficiency of the proposed method in the domain of virtual signal generation, the obtained results were compared with those of CACGAN [10], ML1D-GAN [13], ACGAN [53], and CycleGAN [54]. In this study, data sets consisting of 200 and 400 points were utilized for time domain signals. The results for the various image generation methods are shown in Figure 13.

Subsequently, the method detailed in Section 3.4 was employed to assess the similarity of the generated signals. The accuracy results are listed in Table 5.

As depicted in Table 5, when the number of training samples reached 400, the proposed method demonstrated advantages over the alternative approaches. However, with a limited number of data samples (200), there was a gap in diagnostic accuracy when compared to CACGAN and ACGAN. While CACGAN effectively extracted prominent features from the source image, it struggled with capturing subtle features present in low-amplitude regions. This inconsistency primarily stems from a mismatch between the signal-to-noise ratio and the source image. Similarly, ACGAN, CycleGAN, and ML1D-GAN failed to accurately replicate the signal distribution observed in the original image, thereby inadequately reflecting the mesh and normal signal-to-noise ratio characteristics.

#### 3.5.2. Comparison of DT Methods

1. Comparison of the gear fault

**Figure 14:**
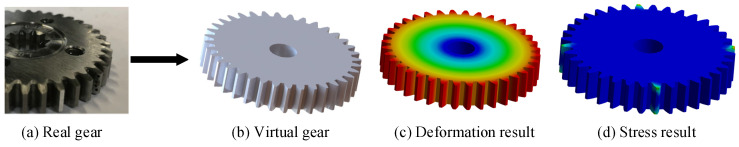
Contrast gear modeling.

This section provides a comparative analysis of fault diagnosis methods leveraging DT modeling [21]. At present, such approaches primarily focus on the individual modeling and analysis of components such as bearings, gears, and gear shafts. The relevant parameters of the objects and the results are provided in Table 6 and Figure 14.

A DT model was applied to investigate the planetary reducer gear operating at a speed of 45 rpm. Our methodology entails modeling and simulating the gear system, subsequently evaluating it to generate virtual signals. These signals can then be compared against real data for validation.

**Figure 15:**
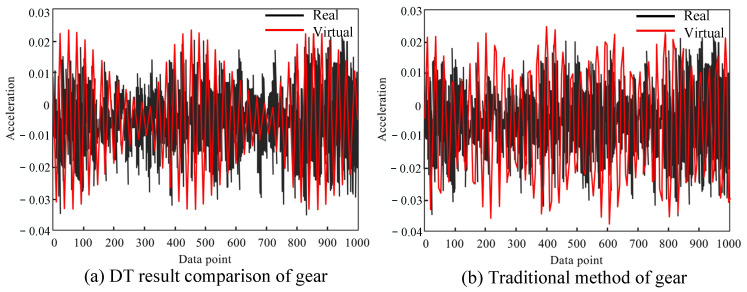
Comparison of gear results.

Figure 14a,b represent the virtual signal generated with the DT and the original signal, respectively. Upon calculating the Hamming distance, values of 2 for Figure 4 and Figure 14a,b were obtained. This comparison highlights the ability of the DT modeling approach to accurately represent real signals, as well as exhibiting its improved signal-to-noise ratio (SNR) and distribution characteristics. (See Figure 15).

2. Comparison of the bearing fault

To expand the fault types and assess the efficacy of the DT model for bearing fault diagnosis, we compared it with the traditional method [55] using the HRB6205 bearing as the basis. The pertinent parameters are elaborated in Table 7.

**Figure 16:**
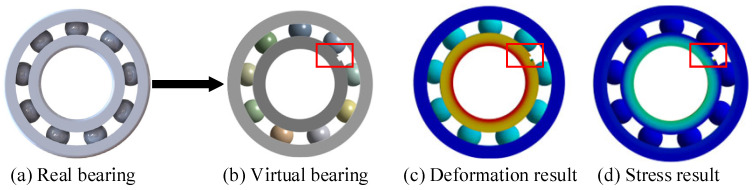
Contrast bearing modeling. Fault is marked by red frame.

The DT model was utilized to construct the bearing fault model, with the operational condition set at 1005 rpm, and the fault type simulated as a crack in the bearing’s inner ring. The outcomes are depicted in Figure 16.

**Figure 17:**
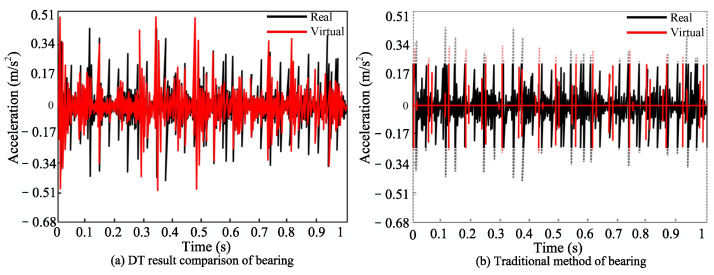
Comparison of bearing results.

The comparison between the proposed and original methods is shown in Figure 17a,b. Through the Hamming distance, the similarity of (a) was found to be 97.3%. This method captures the time-frequency characteristics of the original signal, and highlights its effectiveness in bearing fault diagnosis.

The accuracy of DT models can be significantly enhanced through methods such as data acquisition and integration, dynamic real-time simulation, multi-dimensional optimization, and interactive feedback loops. An accurate representation of entities can be achieved with the DT model, with high-quality data being collected from sensors, operational histories, and environmental conditions. The integrity and accuracy of these data are ensured through sophisticated acquisition and integration techniques, providing essential input for the model. Through dynamic simulation, the capability to monitor and predict faults in real time is enabled for the DT model. It is not confined to optimizing a single objective or function; instead, considerations are made comprehensively across multiple dimensions. The support for human–computer interactions allows for adjustments and optimizations based on insights derived from the model. Moreover, the results from actual operations are fed back into the model, facilitating continuous calibration and optimization. This process forms a closed-loop system that fosters continuous improvement. In essence, these attributes collectively enhance the precision and application effectiveness of the model, showcasing its immense potential for managing, optimizing, and predicting complex systems.

### 3.6. Industrial Trial of the DT Model

**Figure 18:**
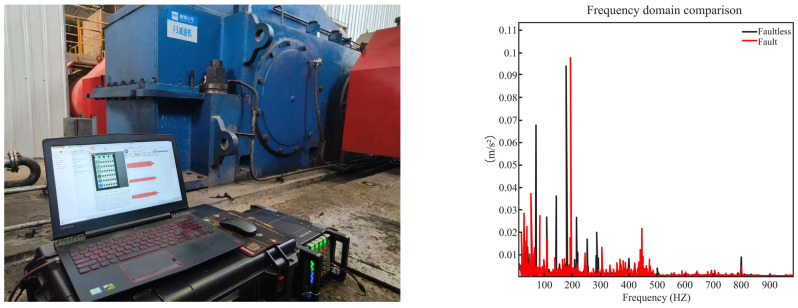
Data comparison.

The DT was applied in the context of industrial production for collaboration with enterprises to test and diagnose faults under real working conditions. In this research, we focused on the DL0509Y reducer. Its specific working conditions are as follows: a maximum input speed of 1200 rpm, noise levels not exceeding 70 dB at a distance of 1.5 m from the box, a reversal time of at least 15 min, and operation duration of 4 h. The results are shown in Figure 18, and the similarity test was carried out using the Differential Hash algorithm and Hamming distance, and the accuracy was 98.95%.

## 4. Human–Computer Interactions

The industrial DT system is a complex system that involves the integration of humans, machines, and the environment, thus presenting various challenges in terms of human–computer interactions. Ensuring safety, facilitating cooperation between humans and machines, and adhering to environment rules are crucial aspects of remote control in human–machine interactions. To achieve virtual–real interactions, it is essential to accurately model the state of the object so that its virtual representation can simulate real-world responses, allowing the virtual and physical worlds to maintain synchronization.

In addition, based on condition monitoring research, the interactions between the equipment were added [56]. First, physical entities are connected to virtual models through Universal Asynchronous Transceiver (UART) serial ports. The collected dynamic information is then transmitted in real time to the Unity3D platform, thus reflecting the reducer’s operational state. The status of the virtual model is continuously updated and the data are stored in a MySQL cloud database. To interact with the database, the js language is used to read and write information. Additionally, front-end HTML files are utilized for description in E-Charts. Finally, a URL link is created in Unity3D to seamlessly integrate the web chart into the platform.

The fault identification model’s calling function is compiled into a C# dynamic link library in MATLAB using the deploy tool toolkit. In Unity3D, the C# language is used to call the dynamic link library and import the collected real-time data. The virtual model displays the operating status and fault alarm signal of the reducer system in real time, transmitting the information to the operator. The sensor provides feedback on the amplitude at the measuring point to the user. The state detection part is connected to an external camera device, enabling real-time monitoring of the fault test platform in the real world. The status information bar displays the feedback result after diagnosis and prediction. The measured data in the system platform could be displayed using E-Charts. The process is shown in Figure 1.

The integration of DT technology for condition monitoring and fault diagnosis has been marked by a pivotal advancement towards intelligent and precise industrial maintenance. Through the creation of a virtual model that mirrors operational states and behavioral traits in real time, fresh insights and approaches are provided for the health assessment and malfunction identification of equipment. In the domain of condition monitoring, the analysis of various parameters such as temperature, noise, and vibration is conducted in real time, accurately reflecting the health of the machinery. This not only increases the monitoring efficiency and precision, but also allows for the anticipation of potential failures.

In the realm of fault diagnosis, good results can be achieved with DT technology through the construction of a precise virtual counterpart that simulates performance across different scenarios. In this way, anomalies can be pinpointed, fault origins can be swiftly traced, and pre-emptive warnings can potentially be offered. Essentially, significant refinement in the processes of condition monitoring and fault diagnosis across industries has been enabled through the use of DT models, ensuring real-time synchronization between the virtual and actual systems, thereby enabling more accurate state evaluation and fault detection.

**Figure 19:**
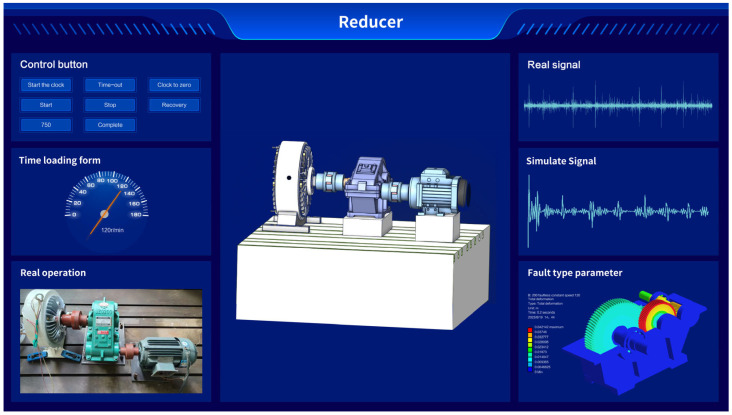
Digital twin system diagram.

The reducer DT system based on Unity3D is depicted in Figure 19. The functional area and control button are located in the upper left corner, next to the loading speed setting interface. In the lower left corner, a real-time interface of the experimental platform is used for state monitoring of the reducer. The real-time vibration signal is displayed in the upper right corner, while the simulation operation module is located beneath it.

## 5. Conclusions

In this research, DT technology was utilized to construct a comprehensive model that compensates for the shortcomings of traditional dynamic modeling and single-part analysis, allowing the issues associated with real-time condition monitoring and fault diagnosis in reducers to be solved. Through incorporating external factors (e.g., environmental and noise), a DT fault diagnosis system was established. Human–computer interaction and online diagnosis were also enabled through the use of Internet of Things (IoT) technology.

With the proposed approach, the limitations of idealized factors in modeling and the lack of 3D visualization in traditional system were overcome, resulting in a more thorough description of the operating state and working conditions. The DT model was integrated with fault diagnosis for real-time condition monitoring and diagnosis of the reducer. Then, its feasibility and accuracy regarding fault diagnosis were verified experimentally. Finally, the DT model was validated through a test in the industrial field and compared with other advanced methods, fulfilling our initial expectations of high accuracy, 3D visualization, and human–machine interaction. The main conclusions of this study are as follows:

(1) The fault diagnosis of the reducer achieved in this work suggests the applicability of DT models for the synchronization of operations in both virtual and real worlds.
(2) The experimental results show that the proposed method can ensure that the error remains within 1%, thus achieving accurate fault diagnosis. Furthermore, the accuracy was 99.5%, providing a reliable foundation for fault diagnosis.
(3) The diagnostic rate in the industrial application under rough working conditions was 98.95%—indisputably lower than that obtained in the laboratory. These results may inspire confidence in DT models for online fault diagnosis of reducers to a certain extent.

## Figures and Tables

**Table 1:** Basic dimensions of high-speed shaft gear.

Code	Meaning	Value	Code	Meaning	Value
*Z*	Number of teeth	22	*C*	Radial clearance coefficient	0.25
*Mn*	Normal module	2.5	*a*	Center to center spacing	125
*α*	Tooth profile angle	20	*T_w_*	Tooth width	30
*ha*	Addendum coefficient	1	*A_g_*	Gear accuracy class	6

**Table 2:** Structural steel material parameters.

Name	Elasticity Modulus	Poisson Ratio	Density
Numerical value	206 GPa	0.3	7850 kg/m^3^

**Table 3:** Calculation results of specific data.

Code	*f_e_* (Hz)	*n* (rpm)	*f_m_* (Hz)	Code	*f_e_* (Hz)	*n* (rpm)	*f_m_* (Hz)
Z_1_	2	120	44	Z_3_	0.57	34.2	11
Z_2_	0.57	34.2	44	Z_4_	0.13	7.62	11

**Table 4:** Similarity test results.

Code	Hamming Distance	Similarity
Faultless constant speed	1	99.9%
Faultless variable speed	1	99.7%
Fault constant speed	2	99.6%
Fault variable speed	4	99.3%
Average	2	99.5%

**Table 5:** Comparison of proposed method with other methods based on image generation.

Method	Measured Data	Accuracy (%)
CACGAN	400	94.32
	200	82.3
ML1D-GAN	400	84.33
	200	73.64
ACGAN	400	88.78
	200	79.69
CycleGAN	400	81.2
	200	73.86
Proposed	400	99.5
	200	76.74

**Table 6:** Basic dimensions of gear.

Meaning	Numerical Value	Meaning	Numerical Value
Number of teeth	36	Gear accuracy class	6
Normal module	1.5	Elasticity modulus	206 GPa
Tooth profile angle	20	Poisson ratio	0.3
Tooth width	30	Density	7850 kg/m^3^

**Table 7:** Basic dimensions of bearing.

Meaning	Numerical Value	Meaning	Numerical Value
Diameter of outer ring	46.98	Pitch diameter	39.04
Diameter of inner ring	31.10	Ball diameter	7.94

## Data Availability

The data that support the findings of this study are available from the corresponding author upon reasonable request.

## Nomenclature

*MO*	The traditional DT model of a reducer	*D_e_*	Noise effect
*I*_1_	Historical data	*C*	Empirical parameters
*I*_2_	Behavioral data	*B*	Empirical parameters
*I*_3_	Morphological data	*η*	Dynamic viscosity
*P*_1_	Device relationship	*L*	Length
*MO_curr_*	Monitor data-driven dynamic update models	*λ*	Linear thermal expansion coefficient
*I*_4_	Online data	*ω_n_*	Noise frequency
*I*_5_	Updated data	*ω_d_*	Natural frequency
*P*_1_ *^curr^*	Current characteristics	*F_ex_*	External force
*M_DT_*	Reducer DT model	*a_n_(t)*	The vibration acceleration under noise
*M_G_*	Geometric model	*G(t)*	Transfer function
*M_A_*	Analytical model	*ω*	Angular velocity
*M_E_*	Environmental model	*ξ*	Damping coefficient of the reducer
*P_g_*	Dynamic model	*Z*	Number of teeth
*R_g_*	Mechanical model	*Mn*	Normal module
*J*	The gear moment of inertia	*α*	Tooth profile angle
*θ*	Rotation angle	*ha **	Addendum coefficient
*T_tor_*	Input torque	*ca **	Radial clearance coefficient
*G*	Transfer ratio	*a*	Center to center spacing
*F_mesh_*	Meshing force	*T_w_*	Tooth width
*K*	Gear stiffness	*A_g_*	Gear accuracy class
*x*	Displacement	*n*	Speed of the gear shaft
*D*	Damping coefficient	*f_e_*	Rotation frequency
*v*	Velocity	*f_m_*	Meshing frequency
*A_l_*	Algorithms	*m*	Mass of each gear shaft
*D_is_*	Discriminator	*d*	Minimum Hamming distance
*k_d_*	Knowledge bases	*n_c_*	Code word length
*h_d_*	Historical data	*k_bit_*	Number of information bits
*T_e_*	Temperature effect		

## References

[1] Asadi E., Li B., Chen I.-M. (2018). Pictobot: A cooperative painting robot for interior finishing of industrial developments. IEEE Robot. Autom. Mag..

[2] McNames J. (2001). Fourier series analysis of epicyclic gearbox vibration. J. Vib. Acoust..

[3] Guo Y.C., Parker R.G. (2011). Analytical determination of mesh phase relations in general compound planetary gears. Mech. Mach. Theory.

[4] Lei Y.G., Zuo M.J., He Z.J., Zi Y.Y. (2010). A multidimensional hybrid intelligent method for gear fault diagnosis. Expert Syst. Appl..

[5] Lebold M., McClintic K., Campbell R., Byington C., Maynard K. Review of vibration analysis methods for gearbox diagnostics and prognostics. Proceedings of the 54th Meeting of the Society for Machinery Failure Prevention Technology.

[6] Bajrić R., Sprečić D., Zuber N. (2011). Review of vibration signal processing techniques towards gear pairs damage identification. Int. J. Eng. Technol..

[7] Liu L., Liu J., Zhou Q., Huang D. (2022). Machine learning algorithm selection for windage alteration fault diagnosis of mine ventilation system. Adv. Eng. Inform..

[8] Xu Y., Li Z., Wang S., Li W., Sarkodie-Gyan T., Feng S. (2021). A hybrid deep-learning model for fault diagnosis of rolling bearings. Measurement.

[9] Chen J. (2018). Industrial robot technology and its typical application analysis. J. Electron. Res. Appl..

[10] Dixit S., Verma N.K., Ghosh A.K. (2021). Intelligent fault diagnosis of rotary machines: Conditional auxiliary classifier gan coupled with meta. learning using limited data. IEEE Trans. Instrum. Meas..

[11] Grieves M.W. (2005). Product life cycle management: The new paradigm for enterprises. Int. J. Prod. Dev..

[12] Ye M., Yan X., Chen N., Jia M. (2023). Intelligent fault diagnosis of rolling bearing using variational mode extraction and improvedone-dimensional convolutional neural network. Appl. Acoust..

[13] Guo Q., Li Y., Song Y., Wang D., Chen W. (2020). Intelligent fault diagnosis method based on full 1-D convolutional generative adversarial network. IEEE Trans. Ind. Inform..

[14] Abdul Z.K., Al-Talabani A.K., Ramadan D.O. (2020). A hybrid temporal feature for gear fault diagnosis using the long short termmemory. IEEE Sens. J..

[15] Yu J., Xu Y., Liu K. (2019). Planetary gear fault diagnosis using stacked denouncing auto encoder and gated recurrent unit neural network under noisy environment and time-varying rotational speed conditions. Meas. Sci. Technol..

[16] Mascaro R., Wermelinger M., Hutter M., Chli M. (2021). Towards automating construction tasks: Large-scale object mapping, segmentation, and manipulation. J. Field Robot..

[17] Lei Y., Lin J., Zuo M.J., He Z. (2014). Condition monitoring and fault diagnosis of planetary gearboxes: A review. Measurement.

[18] Azamfar M., Singh J., Bravo-Imaz I., Lee J. (2020). Multisensor data fusion for gearbox fault diagnosis using 2-D convolutional neural network and motor current signature analysis. Mech. Syst. Signal Process..

[19] Ding A., Qin Y., Wang B., Cheng X., Jia L. (2023). An elastic expandable fault diagnosis method of three-phase motors using continual learning for class-added sample accumulations. IEEE Trans. Ind. Electron..

[20] Ding A., Yi X., Qin Y., Wang B. (2024). Self-driven continual learning for class-added motor fault diagnosis based on unseen fault detector and propensity distillation. Eng. Appl. Artif. Intell..

[21] Ding A., Zhang Y., Zhu L., Li H., Huang L. (2023). Intelligent recognition of rough handling of express parcels based on CNN-GRU with the channel attention mechanism. J. Ambient. Intell. Humaniz. Comput..

[22] Qiao X., Zhang L., Chen C., Yan C., Wang C., Wang W. (2020). Study on transient contact performance of meshing transmission of cycloid gear and needle wheel in RV reducer. J. Eng..

[23] Wang C. (2021). A calculation method of transmission efficiency for RV reducer. J. Eng. Res..

[24] Wu S., Zuo M.J., Parey A. (2008). Simulation of spur gear dynamics and estimation of fault growth. J. Sound Vib..

[25] Shangguan D., Chen L., Ding J. (2020). A digital twin-based approach for the fault diagnosis and health monitoring of a complex satellite system. Symmetry.

[26] Song Z., Shi H., Bai X., Li G. (2023). Digital twin-assisted fault diagnosis system for robot joints with insufficient data. J. Field Robot..

[27] Elsisi M., Tran M.-Q., Mahmoud K., Mansour D.-E.A., Lehtonen M., Darwish M.M. (2022). Effective IoT-based deep learning platform for online fault diagnosis ofpower transformers against cyberattacks and data uncertainties. Measurement.

[28] Grieves M., Vickers J. (2017). Digital twin: Mitigating unpredictable, undesirable emergent behavior in complex systems. Transdisciplinary Perspectives on Complex Systems: New Findings and Approaches.

[29] Barricelli B.R., Casiraghi E., Fogli D.A. (2019). Survey on digital twin: Definitions, characteristics, applications, and design implications. IEEE Access.

[30] Peng P., Wang J. (2019). NOSCNN: A robust method for fault diagnosis of RV reducer. Measurement.

[31] Aivaliotis P., Georgoulias K., Chryssolouris G. (2019). The use of Digital Twin for predictive maintenance in manufacturing. Int. J. Comput. Integr. Manuf..

[32] Errandonea I., Beltrán S., Arrizabalaga S. (2020). Digital twin for maintenance: A literature review. Comput. Ind..

[33] Melesse T.Y., Pasquale V.D., Riemma S. (2020). Digital twin models in industrial operations: A systematic literaturereview. Procedia Manuf..

[34] Wright L., Davidson S. (2020). How to tell the difference between a model and a digital twin. Adv. Model Simul. Eng. Sci..

[35] Lechler T., Fuchs J., Sjarov M., Brossog M., Selmaier A., Faltus F., Donhauser T., Franke J. Introduction of a comprehensive structure model for the digital twin in manufacturing. Proceedings of the 2020 25th IEEE International Conference on Emerging Technologies and Factory Automation (ETFA).

[36] Rasheed A., San O., Kvamsdal T. (2020). Digital twin-values, challenges and enablers from a modeling perspective. IEEE Access.

[37] Bordeleau F., Combemale B., Eramo R., Van Den Brand M., Wimmer M. Towards model-driven digital twin engineering: Current opportunities and future challenges. Proceedings of the First International Conference on Systems Modelling and Management (ICSMM 2020).

[38] Ríos J., Staudter G., Weber M., Anderl R. (2020). Enabling the digital twin: A review of the modelling of measurement uncertainty on data transfer standards and its relationship with data from tests. Int. J. Prod. Lifecycle Manag..

[39] Karve P.M., Guo Y., Kapusuzoglu B., Mahadevan S., Haile M.A. (2020). Digital twin approach for damage-tolerant mission planning under uncertainty. Eng. Fract. Mech..

[40] Andronas D., Kokotinis G., Makris S. (2021). On modelling and handling of flexible materials: A review on digital twins and planning systems. Procedia CIRP.

[41] Matulis M., Harvey C. (2021). A robot arm digital twin utilizing reinforcement learning. Comput. Graph..

[42] He B., Cao X., Hua Y. (2021). Data fusion-based sustainable digital twin system of intelligent detection robotics. J. Clean. Prod..

[43] Jia W., Wang W., Zhang Z. (2022). From simple digital twin to complex digital twin Part I: A novel modeling method for multi-scale and multi-scenario digital twin. Adv. Eng. Inform..

[44] Zhang H., Qi Q., Ji W., Tao F. (2023). An update method for digital twin multi-dimension models. Robot. Comput.-Integr. Manuf..

[45] Zhang Q., Wei Y., Liu Z., Duan J., Qin J. (2023). A Framework for Service-Oriented Digital Twin Systems for Discrete Workshops and Its Practical Case Study. Systems.

[46] Sharma A., Kosasih E., Zhang J., Brintrup A., Calinescu A. (2022). Digital twins: State of the art theory and practice, challenges, and open research questions. J. Ind. Inf. Integr..

[47] Tao F., Qi Q., Liu A., Kusiak A. (2018). Data-driven smart manufacturing. J. Manuf. Syst..

[48] Tao F., Liu W., Zhang M. (2019). Digital twin five-dimensional model and ten major applications. Comput. Integr. Manuf. Syst..

[49] Zong X., Luan Y., Wang H., Li S. (2021). A multi-robot monitoring system based on digital twin. Procedia Comput. Sci..

[50] Liu Y., Ren H. (2022). Acquisition method of evaluation stress for the digital twin model of ship monitoring structure. Appl. Ocean Res..

[51] Li J., Wang S., Yang J., Zhang H., Zhao H. (2023). A Digital T win-Based State Monitoring Method of Gear Test Bench. Appl. Sci..

[52] Wang R., Zhang S., Liu S., Liu W., Ding A. (2023). A bearing fault diagnosis method for high-noise and unbalanced dataset. Smart Resilient Transp..

[53] Shao S., Wang P., Yan R. (2019). Generative adversarial networks for data augmentation in machine fault diagnosis. Comput. Ind..

[54] Zhu J.Y., Park T., Isola P., Efros A.A. Unpaired Image-to-Image Translation using Cycle-Consistent Adversarial Networks. Proceedings of the IEEE International Conference on Computer Vision (ICCV).

[55] Feng K., Xu Y., Wang Y., Li S., Jiang Q., Sun B., Zheng J., Ni Q. (2023). Digital twin enabled domain adversarial graph networks for bearing fault diagnosis. IEEE Trans. Ind. Cyber-Phys. Syst..

[56] Tu D., Zhang Y., Zhu L., Qin Y., Du Y., Liu M., Ding A. (2023). A bistable energy harvester with low base-acceleration and high root mean square output for train bogies: Theoretical modeling and experimental validation. Smart Mater. Struct..

